# Case Report: Guitarist’s cramp as the initial manifestation of dopa-responsive dystonia with a novel heterozygous GCH1 mutation

**DOI:** 10.12688/f1000research.51433.1

**Published:** 2021-05-07

**Authors:** Takafumi Hasegawa, Tatsuhiko Hosaka, Ryuhei Harada, Ichiro Kawahata, Kyoko Hoshino, Naoto Sugeno, Akio Kikuchi, Masashi Aoki

**Affiliations:** 1 Division of Neurology, Department of Neuroscience and Sensory Organs, Tohoku University Graduate School of Medicine, Sendai, Miyagi, 980-8574, Japan; 2 Department of Pharmacotherapy, Graduate School of Pharmaceutical Sciences, Tohoku University, Sendai, Miyagi, 980-8578, Japan; 3 Department of Pediatric Neurology, Segawa Memorial Neurological Clinic for Children, Kanda, Tokyo, 101-0062, Japan

**Keywords:** Guitarist’s cramp, dystonia, task-specific, dopa-responsive, dopamine, Segawa syndrome, GCH1, DYT5a

## Abstract

Dopa-responsive dystonia (DRD), also known as Segawa syndrome, is a phenotypically and genetically heterogeneous group of neurological disorders that typically presents as early-onset lower limb dystonia with diurnal fluctuation, and exhibits a marked, persistent response to levodopa. Heterozygous loss-of-function mutations in the guanosine triphosphate cyclohydrolase 1 (GCH1) are the most common cause of DRD. In addition to the classic form of the disease, there have been a number of studies addressing atypical clinical features of GCH1 related DRD with variable age of onset. This report describes a 37-year-old Japanese male patient with a 10-year history of focal upper limb dystonia that initially emerged as task-specific, guitarist’s cramp. The dystonic symptoms responded very well to levodopa treatment, and genetic analysis identified a novel heterozygous mutation in the C-terminal catalytic domain of GCH1. Insufficient recognition of this treatable condition often leads to misdiagnosis, which causes delays in the patient receiving adequate dopamine replenishing therapy. A diagnostic trial with levodopa should be considered in all patients with relatively young-onset dystonia, whether they have classic features of DRD or not.

## Introduction

Dopa-responsive dystonia (DRD, Segawa syndrome) is a rare movement disorder typically characterized by childhood-onset walking difficulties due to lower limb dystonia, diurnal fluctuation, and dramatic, sustained response to relatively low-dose levodopa treatment ^1^. Striatal dopamine deficiency due to loss-of-function mutations in the guanosine triphosphate cyclohydrolase 1 (GCH1, EC 3.5.4.16) is the most common etiology in the autosomal dominant form of DRD (DYT5a, OMIM 128230), in which incomplete penetrance and variable phenotype are observed ^2^. Owing to its diverse clinical presentations and poor recognition by general practitioners, DRD is under-reported and may be mistaken for other conditions such as cerebral palsy or psychogenic movement disorder. In this report, an unusual, adult case of DRD/DYT5a initially presenting as guitarist’s dystonia is described.

## Case report

The 37-year-old, right-handed Japanese man with a professional career as a guitar player, was referred to our hospital for the reassessment of upper limb dystonia. Upon assessment, no family history of neurological disorders was reported, and he did not use any regular medication before onset. At the age of 27 years, he began to spend most of his time practicing guitar and three months later, he felt difficulty in picking a pick due to excessive wrist extension and intermittent, tremulous finger movement in the right hand. At the age of 29 years, he was diagnosed with having guitarist’s cramp, for which the oral administration of clonazepam (1.5 mg/day) and trihexyphenidyl (6 mg/day) were prescribed. This treatment proved to be ineffective. After switching from guitar to piano, these strange movements transiently disappeared but later reappeared. Meanwhile, the task-specificity of hand dystonia was gradually lost, and the disabling hand dystonia was induced by other daily activities including opening/closing a screw cap bottle and the brushing of his teeth. After five years from the onset, he had considerable difficulty in playing instruments, and finally, he decided to end his musical activities.

**Figure 1.**
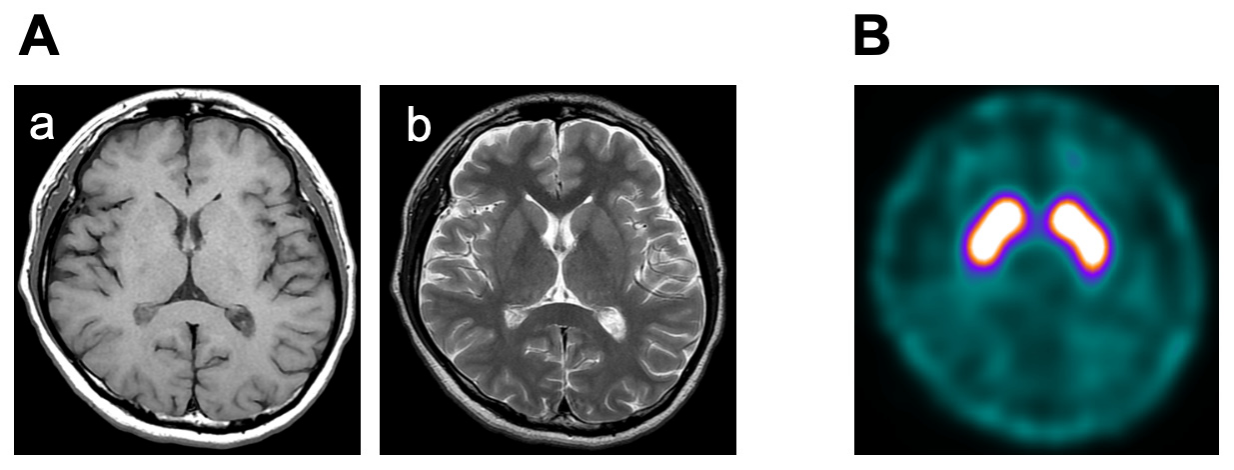
**A**: Cranial magnetic resonance imaging shows no abnormality in (a) T1 and (b) T2 axial sequences. **B**: Transverse dopamine transporter image with ^123^I-β-CIT (2β-carbomethoxy-3β-(4-iodophenyl)tropane) single-photon emission computed tomography demonstrates normal binding of radioligand in both caudate nuclei and putamina.

**Figure 2.**
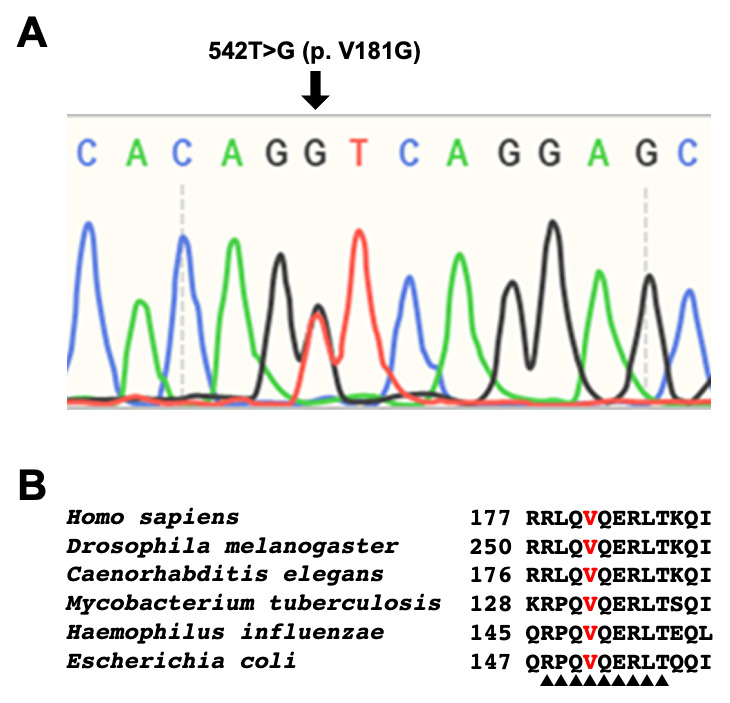
**A**: Sanger sequencing chromatogram which shows a novel heterozygous mutation c. 542T>G (p. Val181Gly) in the first amino acid of exon 5 in the GCH1 gene ( *black arrow*). **B**: Amino acid sequence alignment of the GCH1. Note that the first Val in exon 5 (highlighted in *red*) is highly conserved across species. Residues involved in catalysis are highlighted with *black triangles*

During consultation, neurological examination of cranial nerves, motor function, coordination, sensory function, and autonomic function showed unremarkable results. This was with the exception of action-induced dystonic posturing of the right upper limb with excessive wrist extension and hyperextension of the fingers, though these findings only became prominent during voluntary, skilled movement (Video S1, *Extended data* ^3^). Neither sensory trick nor diurnal variation was observed. Other abnormalities, including cognitive dysfunction, parkinsonism, pyramidal signs, and cerebellar ataxia, were not detected. Workup including electrolytes, renal function tests, complete blood count, liver function tests and urinalysis were unremarkable. In addition, the levels of serum copper and ceruloplasmin were normal. Cranial magnetic resonance imaging and dopamine transporter (DAT) imaging with ^123^I-β-CIT (2β-carbomethoxy-3β-(4-iodophenyl) tropane) single-photon emission computed tomography were normal ( Figure 1). The diagnosis of DRD was suspected since the oral administration of levodopa (300 mg per day) showed dramatic, sustained improvement of the dystonic symptoms on the following day (Video S2, *Extended data* ^3^), and cerebrospinal fluid analyses revealed a significant decrease in homovanillic acid (22.6 ng/ml, normal range: 41.6–178 ng/ml), 5-hydroxyindoleacetic acid (9.1 ng/ml, normal range: 20.0–96.0 ng/ml), and total neopterin (2.0 pmol/ml, normal range: 9.0–20.0 pmol/ml) levels ^4^. The clinical suspicion of DRD was further strengthened by exome analysis and Sanger sequencing showing a novel heterozygous mutation c. 542T>G (p. Val181Gly) in the first amino acid of exon 5 in GCH1 gene ( Figure 2A). The first Val in the 5th exon was located in the enzymatic core of GCH1 at the C-terminus ^5^, and is highly conserved across species ( Figure 2B). This amino acid substitution was predicted to be pathogenic using the *in silico* analysis tools, SIFT and PolyPhen-2. In the half-year follow-up, the patient’s dystonic symptom was well-controlled by the levodopa therapy without any adverse side effects.

## Discussion

In most cases of adult-onset focal limb dystonia, the exact, underlying etiology remains unclear, but in some cases, dystonia occurs due to specific biochemical defects and genetic alterations ^6^. A good example is the DRD caused by genetic defects in dopamine biosynthesis. Deficiency of GCH1, a rate-limiting enzyme in the biosynthetic pathway of tetrahydrobiopterin, is the most common and well-characterized condition that manifests as DRD ^7^. In contrast to the childhood-onset GCH1-related DRD, adult cases often present parkinsonism followed by dystonia, and the movement problems progress slowly without diurnal variation ^8, 9^. Furthermore, a number of studies describing atypical or incompatible features of GCH1 deficient-DRD with variable age of onset exist ^2^. Theoretically, DRD is considered to be a neurotransmitter disorder that is not accompanied by the nigrostriatal degeneration; however, some patients with adult-onset dystonia-parkinsonism or Parkinson’s disease without any dystonic feature carrying GCH1 mutation have been known to show abnormal DAT imaging ^2^.

The most conspicuous feature in this patient’s case is that the dystonic movement which started to present as a guitarist’s cramp. Although infrequent, a few reports of DRD/DYT5a presenting task-specific dystonia in the upper limb have been presented ^10, 11^. Among them, the most common phenotype was writer’s cramp, which became obvious during the disease progression. Conversely, there was only one case report which presented adult-onset guitarist’s cramp in the family carrying GCH1 truncating mutation (p. Arg216stop) ^12^. A genotype-phenotype correlation was unlikely, because even among members of the same family with the same mutation, the movement symptoms were different. While the pathophysiology of task-specific or occupational dystonia still remains elusive, overtraining and genetic predisposition may contribute to the expression of dystonic symptoms. Indeed, about 20% of patients with musician’s dystonia have a family history of the disorder, and a genome-wide association study demonstrated a possible link between musician’s dystonia and the intronic variant in the arylsulfatase G gene ^13^. Furthermore, patients with DYT1 and DYT11 dystonia rarely have writer’s cramp as the cardinal symptom ^14, 15^. Although we did not conduct personality and behavioral assessment of the patient, specific personality traits including susceptibility to anxiety and stress, or perfectionism may also increase the risk of developing dystonia ^16^.

In conclusion, we reported a case of a 37-year-old male who was diagnosed with DRD/DYT5a, which was confirmed through genetic sequencing. The patient presented focal upper limb dystonia which first emerged as task-specific, guitarist’s cramp. The clinical heterogeneity of DRD often makes diagnosis difficult and leads to therapeutic delay. Our experience further underscores the broad clinical presentations of DRD as well as advocating for the diagnostic value of trying levodopa and genetic testing in dystonia.

## Data availability

### Underlying data

All data underlying the results are available as part of the article and no additional source data are required.

### Extended data

Dryad: Dopa-responsive dystonia patient response before and after levodopa treatment. https://doi.org/10.5061/dryad.pzgmsbckd ^3^.

This project contains the following extended data:

- Video S1 (.MOV video of the neurological assessment on admission. Dystonic posturing of the right upper limb with excessive wrist extension and hyperextension of the fingers are seen during the opening of a screw cap bottle. No parkinsonism, pyramidal signs or cerebellar ataxia are observed)
- Video S2 (.MOV video taken on the following day after the oral administration of 300 mg per day of levodopa. The task-specific dystonia in the right upper limb is dramatically improved).

Data are available under the terms of the Creative Commons Zero "No rights reserved" data waiver (CC0 1.0 Public domain dedication).

## Consent

Written informed consent for publication of the clinical details, diagnostic images and videos was obtained from the patient.
