# Supplementary material for: Case Report: Guitarist’s cramp as the initial manifestation of dopa-responsive dystonia with a novel heterozygous GCH1 mutation
Source: F1000Res. 2021 May 7;10:361. [Version 1] doi: 10.12688/f1000research.51433.1 (PMC8356262; doi:10.12688/f1000research.51433.1)
Supplement: Peer review history

# Peer review: Reviewer response for version 1

**DOI:** 10.5256/f1000research.54602.r90521

**Published:** 2021-08-17

**Authors:** Juei-Jueng Lin

**Affiliations:** 1 Department of Neurology, Chushang Show-Chwan Hospital, Nantou, Taiwan

Hasegawa T *et al. *report a young adult man with a 10-year history of upper limb dystonia that mimicked as task-specific dystonia. His dystonia responded well to Levodopa treatment and his genetic study revealed a novel heterozygous mutation of GCH1 gene. Therefore, they conclude task-specific dystonia can also be one phenotype of Segawa syndrome or dopa-responsive dystonia (DRD). Meanwhile, a series of neuroimaging studies of this case including CT, MRI and SPECT of the brain were all negative results.

 I am interested in this case, but I have three major comments on this paper:

1. The authors emphasized that the patient presented guitarist’s cramp in his early stage of disease, three months later after an intensive practice of guitar. Meanwhile, they also described the dystonia can also appear in playing other instruments and in some daily activity. This manifestation is different from the disease course of idiopathic task-specific dystonia. The authors should discuss the differences between idiopathic task-specific dystonia and genetic related task-specific dystonia.
2. The patient responded well to treatment with levodopa and was subsequently diagnosed to be DRD, but did not have a family history. The genetic testing for GCH1 gene revealed a novel heterozygous mutation of c. 542T>G. However, the paper does not mention genetic analysis for GCH1 of his parent or siblings. Therefore, the authors should provide the genetic testing of GCH1 of other families to confirm genetic basis of their reported patient, In addition, they also can discuss the intrafamilial variability of photype in DRD family.
3. The authors also reported a normal presynaptic nigrostriatal dopamine function by a normal SPECT study in their DRD case. There have been reports in literature with an abnormal presynaptic nigrostriatal dopamine function in DRD patient presented with either dystonia or Parkinsonism. The author should discuss the relationship of presynaptic dopamine function in patients with DRD presented with variably clinical phenotypes.

Are enough details provided of any physical examination and diagnostic tests, treatment given and outcomes?

Partly

Is the case presented with sufficient detail to be useful for other practitioners?

Partly

Is sufficient discussion included of the importance of the findings and their relevance to future understanding of disease processes, diagnosis or treatment?

Partly

Is the background of the case’s history and progression described in sufficient detail?

Partly

Reviewer Expertise:

Parkinson's disease and related disorders

I confirm that I have read this submission and believe that I have an appropriate level of expertise to confirm that it is of an acceptable scientific standard, however I have significant reservations, as outlined above.

# Peer review: Reviewer response for version 1

**DOI:** 10.5256/f1000research.54602.r90523

**Published:** 2021-08-09

**Authors:** Taku Hatano

**Affiliations:** 1 Department of Neurology, Faculty of Medicine, Juntendo University, Tokyo, Japan

Hasegawa and colleagues reported the case with DYT5, who exhibited the focal dystonia mimicking musician's cramp. They described the patient’s medical story and assessed the patient’s state by appropriate examinations, including neurological examination, cranial MRI, DAT scan, and genetic analysis. They also mentioned the important learning points. Thus, I am interested in this paper.

 But I have a comment on this paper.

**Major point:**

- The authors pronounced that in the early stage, the patient exhibited the mimicking guitarist’s cramp, but his dystonia emerged in not only task-specific but also usual situations. This finding is different from idiopathic task-specific dystonia in the disease course. The authors should discuss the difference between genetic-related and idiopathic task-specific dystonia.

Are enough details provided of any physical examination and diagnostic tests, treatment given and outcomes?

Yes

Is the case presented with sufficient detail to be useful for other practitioners?

Yes

Is sufficient discussion included of the importance of the findings and their relevance to future understanding of disease processes, diagnosis or treatment?

Yes

Is the background of the case’s history and progression described in sufficient detail?

Yes

Reviewer Expertise:

Parkinson's disease, Movement disorders

I confirm that I have read this submission and believe that I have an appropriate level of expertise to confirm that it is of an acceptable scientific standard.

# Peer review: Reviewer response for version 1

**DOI:** 10.5256/f1000research.54602.r84972

**Published:** 2021-06-08

**Authors:** Hiroshi Ichinose

**Affiliations:** 1 School of Life Science and Technology, Tokyo Institute of Technology, Yokohama, Japan

The authors described an adult male case of DRD/DYT5a who initially developed focal upper limb dystonia and diagnosed as guitarist’s dystonia, and found that the dystonic symptoms responded very well to levodopa treatment. They propose that a diagnostic trial with levodopa should be considered in all patients with relatively young-onset dystonia regardless of whether they have typical features of DRD or not.

 This paper is worthwhile publishing to report that adult-onset focal limb dystonia can be presented in patients with DRD/DYT5a, suggesting a broad clinical presentation of DRD/DYT5a.

 The reviewer would like to ask for the following minor revision to the manuscript;

1. The authors should discuss the benefits and possible adverse effects of levodopa treatment for the patients with dystonia, whereas the authors propose the diagnostic value of levodopa treatment.
2. The biochemical analysis of HVA and neopterin in the CSF is beneficial for a differential diagnosis of DRD/DYT5a from other types of dystonia/parkinsonism. It should be discussed clearly in the text.

Are enough details provided of any physical examination and diagnostic tests, treatment given and outcomes?

Yes

Is the case presented with sufficient detail to be useful for other practitioners?

Yes

Is sufficient discussion included of the importance of the findings and their relevance to future understanding of disease processes, diagnosis or treatment?

Yes

Is the background of the case’s history and progression described in sufficient detail?

Yes

Reviewer Expertise:

Biochemistry

I confirm that I have read this submission and believe that I have an appropriate level of expertise to confirm that it is of an acceptable scientific standard.
